# Cognitive dysfunction in spinocerebellar ataxias

**DOI:** 10.1590/S1980-57642009DN30300002

**Published:** 2009

**Authors:** Helio Afonso Ghizoni Teive, Walter Oleschko Arruda

**Affiliations:** 1 Movement Disorders Unit, Neurology Service, Internal Medicine Department, Hospital de Clínicas, Federal University of Paraná, Curitiba, PR, Brazil.

**Keywords:** Spinocerebellar ataxia, cognitive dysfunction, dementia

## Abstract

Spinocerebellar ataxias (SCAs) comprise a heterogeneous group of complex neurodegenerative diseases, characterized by the presence of progressive cerebellar ataxia, associated or otherwise with ophthalmoplegia, pyramidal signs, extrapyramidal features, pigmentary retinopathy, peripheral neuropathy, cognitive dysfunction and dementia.

**Objective:**

To verify the presence of cognitive dysfunction among the main types of SCA described in the literature.

**Methods:**

the review was conducted using the search system of the PUBMED and OMIM databases.

**Results:**

Cognitive dysfunction occurs in a considerable proportion of SCA, particularly in SCA 3, which is the most frequent form of SCA worldwide. Dementia has been described in several other types of SCA such as SCA 2, SCA 17 and DRPLA. Mental retardation is a specific clinical feature of SCA 13.

**Conclusions:**

The role of the cerebellum in cognitive functions has been observed in different types of SCAs which can manifest varying degrees of cognitive dysfunction, dementia and mental retardation.

## Abstract (pt)

**Fundamento:**

As ataxias espinocerebelares (AECs) compreendem um grupo heterogêneo de enfermidades neurodegenerativas, que se caracterizam pela presença de ataxia cerebelar progressiva, associada de forma avariada com oftalmoplegia, sinais piramidais, extrapiramidais, retinopatia pigmentar, neuropatia periférica, disfunção cognitiva e demência.

**Objetivo:**

Revisar a presença de disfunção cognitiva entre os principais tipos de AECs publicados na literatura mundial.

**Métodos:**

A revisão foi realizada através da pesquisa pelo sistema do PUBMED e do OMIM.

**Resultados:**

Disfunção cognitiva ocorre em uma parcela considerável de AECs, particularmente na AEC 3, que é a forma de AEC mais encontrada em todo o mundo. Demência também tem sido descrita em alguns tipos de AECs como a AEC 2, a AEC 17 e a DRPLA. Retardo mental é característica clínica peculiar da AEC tipo 13.

**Conclusões:**

O papel do cerebelo nas funções cognitivas vem sendo enfatizado cada vez mais e diferentes tipos de AECs, apresentam graus variados de disfunção cognitiva, demência e também retardo mental.

Spinocerebellar ataxias (SCAs) are a large group of complex and heterogeneous hereditary neurodegenerative diseases characterized by cerebellar ataxia, associated or otherwise to varying degrees with ophthalmoplegia, pyramidal and extrapyramidal features, dementia, epilepsy, pigmentary retinopathy, motor neuron disease and peripheral neuropathy.^1-7^

The onset of SCA typically occurs at between 30 and 50 years old in most forms, although some cases may present initial clinical signs by the age of 20 years or beyond 60 years of age.^1-3^

The degenerative process involves the cerebellum and its efferent and afferent connections, and may affect other central nervous system structures including basal ganglia, brainstem nuclei, pyramidal tracts, posterior spinal cords, and spinal cord anterior horns.^1-9^

Neuroimaging, including MRI, shows the presence of cerebellar atrophy, associated with brainstem atrophy (olivopontocerebellar atrophy) or otherwise.^2-10^

Some patients may also present cerebral atrophy.^6^

Van de Warrenburg et al. (2002) assessed the prevalence of autosomal dominant cerebellar ataxias and found, on average, 3 cases per 100,000 inhabitants.^11^

Harding classified autosomal dominant cerebellar ataxia (ADCA) into 4 basic clinical types, defined below:

- Type 1 characterized by optic atrophy, ophthalmoplegia, dementia, amyotrophy and extrapyramidal signs.
- Type 2 with retinal degeneration, which may be associated with ophthalmoplegia and extrapyramidal signs.
- Type 3 a form of “pure cerebellar ataxia” and
- Type 4 associated with deafness and the presence of myoclonus.^12^

Great advances in molecular genetics using the PCR technique (polymerase-chain reaction) have led to several genetic loci and genes being identified in different chromosomes, thus allowing the use of a more rational classification based on clinical and genetic data.^1-3,9,10,13-20^

The main objective of this article was to review the literature on the presence of cognitive dysfunction and dementia in patients with SCA.

## Methods

We reviewed the articles published in the last 5 years on SCA, placing special emphasis on those reporting the presence of cognitive dysfunction and dementia. The main databases used were the PUBMED and OMIM (Online Mendelian Inheritance in Man) of the John Hopkins University.

## Results

### Spinocerebellar ataxias – general overview

Table 1 shows the main known types of SCA and depicts the genetic loci, genes, mutations and related proteins.

**Table 1:** Spinocerebellar ataxias: summary of genetic defects.

SCA	Chromosome	Gene	Mutation	Protein
SCA 1	6p22.3	ATAXIN1	CAG	Ataxin 1
SCA 2	12q24.13	ATAXIN2	CAG	Ataxin 2
SCA 3	14q32.12	ATAXIN3	CAG	Ataxin 3
SCA 4	16q24-qter	SCA4	(PLEKHG4) ?	-
SCA 5	11q13.2	SPTBN2	D/MM	Beta-III Spectrin
SCA 6	19p13.13	CACNA1A	CAG	CACNA1A
SCA 7	3p14.1	ATXN7	CAG	Ataxin.7
SCA 8	13q21	KLHLIAS	CTG	Kelch-like 1
SCA 9	Reserved	-	-	-
SCA 10	22q13.31	ATXN10	ATTCT	Ataxin.10
SCA 11	15q14-q21.3	SCA11	-	-
SCA 12	5q32	PPP2R2B	CAG	PPP2R2B
SCA 13	19q13.33	KCNC3-	MM	KCNC3
SCA 14	19q13.42	PRKCG	MM	PRKCG
SCA 15	3p24.2-3ptr	ITPR1	PM	-
SCA 16	8q23-q24.1	-	-	-
SCA 17	6q27	TBP	CAG	TBP
SCA 18	7q31-q32	-	-	-
SCA 19	1p21-q21	-	-	-
SCA 20	11		-	-
SCA 21	7p21.3-p15.1	-	-	-
SCA 22	1p21-q23	-	-	-
SCA 23	20p13-p12.2	-	-	-
SCA 24	Reserved	-	-	-
SCA 25	2p21-p15	-	-	-
SCA 26	19p13.3	-	-	-
SCA 27	13q33.1	FGF14	MM	FGF14
SCA 28	18p11.22-q11.2	-	-	-
SCA 29	3p26		-	-
SCA 30	4q34.3-q35.1	-	-	-
DRPLA	12p13.31	ATN1	CAG	Atrophin 1
EA 1	12p13	KCNA1	PM	K Channel
EA 2	19p13	CACNA-1A	PM	Ca Channel
EA 3	1q42	-	-	-
EA 4	-	-	-	-
EA 5	2q22-q23	CACNB4	PM	Cav2.1
EA 6	5p	SLCIA3	PM	EAAT1

SCA, spinocerebellar ataxia; DRPLA, dentatorubral-pallidoluysian atrophy; D, deletion; MM, "missense" mutation; PM, point mutation; EA, episodic ataxia.

From a total of 30 different known loci, SCA 1, 2, 3, 6, 7, 17 and dentatorubral pallidoluysian atrophy (DRPLA) are caused by mutations characterized by the presence of expanded and unstable polymorphic trinucleotide CAG repeats in the gene encoding region. The product of the gene is a protein called ataxin. This protein contains amino acid groups and these diseases are known at present as polyglutamine diseases.^10,15-18,20,21^ Some proteins are called ataxin, but the SCA 6 gene is CACNA1A, SCA 17 is a TATA binding protein (TBP) and the DRLPA gene is atrophin-1. The mutant protein, ataxin, has a toxic function that triggers the degenerative process, with the formation of nuclear inclusions in Purkinje cells of the cerebellum, having clear involvement of the ubiquitin proteasome pathway and the protein system known as chaperones. In sum, the polyglutamine-related neurodegenerative diseases are characterized by expansion of polyglutamine within the mutant protein causing the disease. The expression of the mutant protein induces a progressive loss of neuronal function and subsequent neurodegeneration of a specific group of neurons for each form of the disease.^1-9,12-14,16,19,21^

A particular feature of these types of SCA is the anticipation phenomenon, i.e. in successive generations of affected families the onset of the clinical disease occurs at an earlier age and presents with increasingly severe symptoms. The anticipation phenomenon is related to an increase in the number of expansions of the CAG triplet repeat (the larger the expansion, the earlier the age of onset).^2,3,5-10,13,18,19,21^

Another group of SCAs, which includes the types SCA 8, 10 and 12, are caused by a repeat expansion located outside the coding region of genes related to disease which disrupts gene expression.^2-10,13,14,16-19,21^ SCA 8 is associated with a CTG expansion while SCA 10 is caused by pentanucleotide ATTCT expansion which leads to a dysfunction in the protein phosphatase activity of the enzyme known as PP2 which acts on the Purkinje cells.^2-10,13,14,16-19,21^

SCA12 is associated with CAG repeat mutations in the PPP2R2B gene.^22^

A third group of SCAs comprising SCA 5, 11, 13,14, 15 and 27 are caused by different mechanisms and lead to changes in the composition of amino acids (beta-Spectrin III), tau tubulin kinase (TTBK2), potassium channels (KCNC3), protein kinase C (PRKCG), type 1 inositol 1,4,5-triphosphate receptor (ITPR1) and fibroblast growth factor 14 (FGF14), respectively.^2,3,17-20^

Finally, there is a fourth group of SCAs. The gene location of this group has been identified, but the gene mutation and mechanism of disease remain unclear or unknown (SCA 4, 18, 19, 20, 21, 22, 23, 25, 26, 28, 29 and 30).^2,3,10,16-21^

It should be noted that SCA 9 and 24 have yet to be defined (reserved). However, SCA types 19 and 22 are probably allelic forms of the same gene, as are SCA 29 and 15. The SCA 15 and 16 may represent the same disease.

The mutation in the gene for puratrophin, known as the PLEKHG^4^ gene (Chromosome 16q22) is associated with the pure cerebellar ataxia form of Japanese SCA 4 (which is classically characterized as sensory axonal neuropathy), and now also with Japanese dominant ataxia caused by the PLEKHG4 mutation, which has been reassigned as SCA R4. ^2,3,10,16-21^

The neuropathological degenerative process has been studied in depth using mice models of SCA and transgenic Drosophila models.^2,3,6-10,13,14,19,21^

Table 2 lists the main SCAs that may involve cognitive dysfunction or dementia, those associated with mental retardation and finally SCAs which currently have no clearly defined cognitive dysfunction.

**Table 2:** Cognitive function in spinocerebellar ataxias.

Cognitive function	Spinocerebellar ataxias (SCAs)
Cognitive dysfunction	SCAs1, 2, 3,6,7,8,10,12,17, 19,21, DRPLA
Dementia	SCAs 2, 3(?), 8,12,19, DRPLA
Mental retardation	SCA13
Normal cognitive function	SCAs 4,5,11,14,15,16,18,20, 22,23,24,25,26,27,28,29, EA 1 and 2

SCA, spinocerebellar ataxias; DRPLA, dentatorubral-pallidoluysian atrophy; EA, episodic ataxias.

### Spinocerebellar ataxia type 3

SCA 3 is the most common SCA worldwide, while other forms such as SCA 1, SCA 2, SCA 6, SCA 7 and SCA 8 also have a wide prevalence range depending on the ethnic background of the studied population.^1-5, 15,17,23-27^ In a southern Brazilian series of 100 SCA families, the mutation was identified in two-thirds of the cases. SCA 3 is the most frequent (73.5%), SCA 10 represents the second most common (11.8%), followed by SCA 2 (7.4%), SCA 7 (4.4%), SCA 1 (2.9%), and SCA 6 (1.5%).^25^

SCA 3 or Machado-Joseph disease (MJD) has been described as the most commonly found form of SCA in molecular genetics studies worldwide.^1-9,13-15,18-21,25-27^

The disease is characterized by the presence of an expansion of a CAG triplet, at the level of chromosome 14 (32,12), having a range of between 56 to 86 trinucleotíde repeats (normal alleles=12–47).^2,3,10,16-21^

Neuropathological examination shows neuronal loss associated with reactive gliosis in the following structures: substantia nigra, dentate nucleus of the cerebellum, red nucleus, pontine nuclei, other cranial nerve nuclei, Clarke columns, and anterior horn cells of the spinal cord. Globus pallidus may also be involved. Involvement of bulbar olives and both cerebellar and cerebral cortex are also common.^1-9^

Studies on neuroimaging show the presence of cerebellar atrophy, pons atrophy, usually without involvement of olives. A study by Murata and colleagues published in 1998 which employed MRI, showed the presence of cerebellar atrophy, pons, globus pallidus, and the frontal and temporal lobes.^3,4,28^

MJD presents a variable clinical picture including cerebellar ataxia, associated with pyramidal signs, peripheral amyotrophy, nystagmus, eyelid retraction and ophthalmoparesis (“bulging eyes”), facial and tongue fasciculations, eventually extending to the limbs, and the occasional presence of dystonia and parkinsonism. ^2-7,29-32^

In 1980, Lima and Coutinho proposed the following diagnostic criteria for MJD:

- Autosomal dominant inheritance.
- Major neurological signs: cerebellar ataxia, pyramidal signs, extrapyramidal signs and amyotrophy.
- Minor neurological signs: progressive external ophthalmoplegia, dystonia, fasciculations and “bulging eyes”.^30^

In 1992, Paula Coutinho, in his doctoral thesis, updated the diagnostic criteria for MJD as follows:

- Autosomal dominant.
- Onset early in adulthood,
- Presence of supranuclear ophthalmoparesis + ataxia, pyramidal signs, extrapyramidal, with peripheral nervous system involvement.
- Minors signs: fasciculations + eyelid retraction.
- Preservation of higher cortical functions.
- Median survival of 21 years.^31^

Sequeiros and Coutinho conducted a study involving clinical and epidemiological evaluation of 143 patients with SCA 3 and found mild loss of memory in only 2 cases.^32^ MJD is a form of SCA that does not manifest dementia, though the presence of cognitive dysfunction has been described, including executive dysfunction as well as dysfunction in some emotional patients.^33^ In this study, six patients with SCA 3 were tested using a battery of neuropsychological tests, and relative impairment on timed verbal attention tasks and verbal fluency (Stroop, Symbol Digit modalities Oral, and Controlled Oral Word Association test) was found. Executive impairment was seen on the Wisconsin Card Sorting test.^33^

In 2002, Ishikawa et al. published a report of four patients with SCA 3 and a picture of dementia and delirium. The authors reported that the symptoms of dementia and delirium occurred at later stages of the disease and were related not to loss but dysfunction of cerebrocortical neurons, based on neuropathological findings in two patients.^34^ Similarly, Ikeda et al. reported an atypical case of SCA 3 presenting retinal degeneration and dementia.^35^

In routine clinical practice however, most patients with SCA 3 do not present dementia. Maruff et al. evaluated the presence of cognitive deficits in six patients with MJD, using a series of subtests from the Cambridge Neuropsychological Test Automated Battery. These patients presented visual attentional function deficits.^36^ Kawai et al. also assessed cognitive impairment in 16 genetically confirmed MJD and found that these patients had visual and verbal memory deficits, visuospatial and constructional dysfunction and verbal fluency deficits, all of which were unrelated to CAG repeat length.^37^

### Spinocerebellar ataxia type 10

SCA 10 was originally described in families of Mexican origin and has a well-defined clinical picture, namely: the cerebellar syndrome patients have a “pure” cerebellar form, often accompanied by epilepsy and peripheral neuropathy.^25,38,39^ The disease-causing mutation of SCA 10 is a large expansion of the pentanucleotide (ATTCT) repeat located in an intron of a gene of unknown function, SCA10, on chromosome 22q.^25,38,39,40,41^

The DNA test for SCA type 10 detects the size of expanded alleles which can vary between 800 ATTCT and 4500 ATTCT repeats, and yields 100% sensitivity and specificity (with analysis by PCR and Southern blot).^40-42^

SCA 10 typically shows inverse correlation between age of onset of SCA and size of the repeat expanded ATTCT.^40-42^

Neuroimaging studies, particularly MRI, reveal the presence of pan-cerebellar atrophy without abnormalities in other regions.^25,38,39^ There are no known published data on neuropathological examination in SCA 10 although there are studies on experimental SCA 10 models, but the mechanism of the disease with the expansion ATTCT is largely unknown.^25,38,39^

Rasmunssen et al. (2001) published a seminal study involving the clinical and genetic analysis of 4 Mexican families with SCA 10 (eighteen affected members). The affected patients had a mean age of disease onset of 26.7 years (ranging from 14 to 44 years of age) while the number of repetitions of ATTCT ranged from 920 to 4140. The authors observed no significant correlation between the course or onset of disease and number of repeated ATTCT triplets. Clinical data, besides cerebellar ataxia and epilepsy (found in 72.2% of cases) also included peripheral polyneuropathy in 66% of cases (confirmed by nerve conduction studies), predominant cerebellar atrophy on MRI examinations, and in some cases mild pyramidal signs, ocular dyskinesia, cognitive dysfunction and/or behavioral disorders, liver and heart dysfunction, and hematological abnormalities.^38^

To date, SCA 10 has been found only in patients with Mexican ancestry.

In 2002, Matsuura and colleagues investigated the presence of the SCA10 mutation type in non-Mexican populations (white North American, Franco-Canadian, Italian, Spanish and Japanese patients) and found no additional SCA10 cases.^43^ Similarly, Fujigasaki and colleagues failed to detect the presence of the SCA10 mutation type in 10 patients originating from France.^44^

Teive et al., in 2004, published a study on 5 Brazilian families with confirmed diagnosis of SCA 10 who harbored a different phenotype, a form of pure cerebellar ataxia and epilepsy with or without peripheral neuropathy. Approximately 70% of households in Brazil have associated indigenous origins.^25^ In this series of 28 Brazilian patients, seven patients underwent neuropsychological tests (Wechsler Adult Intelligence Scale III, Wechsler Memory Scale III, Controlled Oral Word Association Test instruments, the Mini-Mental State Examination-MMSE and the Hamilton test). Neuropsychological tests were normal, except in one patient who had a low IQ of 73 and mild memory difficulties. The Hamilton test and MMSE were normal in all patients tested.^25^

### Other forms of SCA

SCA 2 is characterized by cerebellar ataxia (with atrophy of the cerebellum on neuroimaging exams) associated with dysarthria, tremor, deep hypo / areflexia of the upper and lower limbs (defining the presence of associated peripheral neuropathy), facial fasciculations and the presence of slow saccadic eye movements.^2-6,19^ Other clinical manifestations include the presence of dystonia, chorea, parkinsonism, myoclonus and dementia.^2-6,19^

Neuropathological findings in SCA 2 include cerebellar atrophy, with loss of Purkinje cells and granular cells, neuron loss in the olivary nucleus, the substantia nigra and anterior horn cells of the spinal cord.^3,4^

The SCA 2 mutation locus is located on chromosome 12, at position 12q 24.13 leading to an expansion of trinucleotide CAG, with between 34 and 59 repetitions (normal alleles=14-32).^2-6,19^

Cognitive deficits have been described in between 5 and 19% of patients with SCA2.^45^

Burk et al., in 1999, performed a study on cognitive deficits in SCA 2. Cognitive function was evaluated in 17 patients with genetically confirmed SCA 2, and in 15 controls, using a neuropsychological test battery including IQ, attention, verbal and visuospatial memory and executive function tests. Twenty-five percent of the SCA2 patients showed evidence of dementia and even in non-demented SCA2 patients there was evidence of verbal memory and executive dysfunction. There was no relationship between test performance and motor disability, age or repeat length at onset.^45^

More recently, Ramocki et al. described a case of SCA 2 presenting with cognitive regression in childhood.^46^

Cases of cognitive dysfunction and even dementia involving other forms of SCA, such as SCA 1, SCA 6, and SCA 8 have been described.^47-51^

Cognitive dysfunction has been observed in between 5–25% of patients at advanced stages of SCA 1.52 Burk et al. defined the presence of executive dysfunction in patients with SCA 1 as the result of impairment of afferent and efferent connections between the prefrontal cortex and the cerebellum, and executive dysfunction within the rank of the “frontal-subcortical dementia”.^47^ Kawai et al., studied thirteen genetically confirmed SCA 6 patients by applying neuropsychological tests and SPECT, and found prefrontal hypoperfusion and cognitive dysfunction in these patients.^48^

Cognitive dysfunction and even dementia has been described more rarely in SCA 12, 17, 19 and 21 as well as in DRPLA.^3,53-57^

SCA 17 is a rare form of autosomal dominant neurodegenerative disease caused by expansion of a CAG triplet repeat in the gene structure of the protein linked to TATA, called the TBP gene (located on chromosome 6q27), which is a factor of initial transcription.^2-4,54,55^ The disease was first described in Japan by Koide et al. in a 14 year-old female patient with clinical onset at 6 years of age, ataxia syndrome, and subsequent intellectual deterioration. Parkinsonism and deep hyper reflexia may also appear during the course of the disease.^54,55^ More recently, many proven cases of SCA type 17 with different clinical presentations have been described, particularly those with a phenotype suggestive of Huntington disease.^58^

SCA 13 is an unusual form of SCA related to chromosome 19q13.33 and mutation in the gene set KCNC3^2-4,7,9,18,19^ that is characterized clinically by the presence of cerebellar ataxia with childhood-onset and which is slowly progressive, associated with dysarthria, moderate mental retardation and delayed motor development motor.^2-4,6-9,18,19^

## Discussion

Cognitive dysfunction or dementia has been described in many forms of SCA and the presence of these clinical manifestations, together with the classical signs of cerebellar motor syndrome, may conceivably be explained by disruption in cerebrocerebellar circuitries at various levels in the central nervous system.^59^

The contribution of the cerebellum to higher cortical functions emerged as a concept following the studies by Schmahmann in 1991.^60^ The author studied the cerebellum, its afferent and efferent connections, particularly via corticopontocerebellar pathways, and their relationship with the cortical association areas.

In 1997, Schmahmann and Sherman studied 20 patients with disease confined to the cerebellum using neurological examinations, and performed bedside mental status tests, neuropsychological studies and anatomical neuroimaging, defining the so-called cerebellar cognitive affective syndrome (CCAS).^61^ This syndrome is characterized by:

(1) disturbances in executive function, including poor planning, perseveration of shifting set, abstract reasoning, and verbal fluency;
(2) visual-spatial disorganization and impaired visual-spatial memory;
(3) personality change characterized by a flattening or blunting affect, and disinhibited, inappropriate, or “giddy” behavior;
(4) difficulty in interpreting and producing logical sequences, and
(5) dysprosodia including language difficulties, mild anomia, and agrammatism.^61^

All these deficits described in the syndrome point to disruption of cerebellar modulation in the neural circuits that link prefrontal, posterior parietal, higher temporal and limbic areas with the cerebellum.^62^

The CCAS is more relevant in patients with lesions involving the posterior lobe of the cerebellum and vermis. Lesions of the anterior lobe of the cerebellum produce only minor changes in executive and visual-spatial functions.^62^

In 2004, Schmahmann formulated a new concept related to the CCAS, which he called dysmetria of thought. Thus, in patients with cerebellar disease there is a component of cognitive and psychiatric CCAS, associated with motor ataxia (thought dysmetria hypothesis).^63^ Patients with cerebellar ataxia have cognitive impairment when the sensorimotor CCAS is involved, affecting the lateral hemisphere of the posterior cerebellum (involved in cognitive processing) or the vermis (limbic cerebellum).^63^

There is converging evidence from neuroimaging, neuropsychological and neuroanatomical studies suggesting the cerebellum contributes to executive control, in particular with respect to verbal working memory as one of the components of the heterogeneous, multifaceted concept of ‘executive function’.^64^

Burke et al. studied the cognitive deficits in 36 patients with SCA type 1, 2, and 3, and compared them with a control group of 8 patients. All patients underwent a neuropsychological test battery that comprised tests of IQ, attention, executive function, verbal and visuospatial memory.^52^ The authors found prominent executive dysfunction in SCA1, and mild deficits of verbal memory in SCA1, SCA2 and SCA3. The authors also stated that the cognitive deficits found could have stemmed from disruption in the cerebrocerebellar circuitry, probably at the pontine level.^52^

Recently, Garrard et al. assessed cognitive and social cognitive functioning in SCA.^65^ The authors studied 15 patients, nine SCA 6 patients and six SCA 3 patients. They used a common test battery which assessed general, social and emotional cognition, including memory, language, visuo-spatial skills, calculation, attention and executive functions, emotional processing and theory of mind (ToM). The results showed that deficits in memory and executive function were more prominent in patients with SCA 3, and that both groups had poor performance on theory of mind (ToM). Based on these results, the authors also commented on the role of the cerebellum in autism.^65^

Brandt et al. compared 21 patients with Huntington’s disease (HD) against 31 patients with cerebellar degeneration, and 29 normal adults, screening for the presence of cognitive dysfunction.^66^ The authors found cognitive deficits in patients with cerebellar atrophy to be less severe than in patients with HD. Cerebellar atrophy patients had greater impairment of executive functions, suggesting a form of subcortical dementia, while patients with HD showed greater spatial deficits and memory impairment as well as executive dysfunction.^66^

Overall, we can conclude that the presence of cognitive dysfunction has been studied in various types of SCAs, especially in SCA 3 due to its higher frequency worldwide. Cognitive dysfunction has also been found in other types of SCA albeit at a lower frequency. Dementia has been described largely in SCA 2, SCA 17 and DRPLA. Finally, SCA type 13 is characterized by the association of cerebellar ataxia and delayed neuropsychomotor development and mental retardation.
